# Comparison of methods for sensitivity correction in Talbot–Lau computed tomography

**DOI:** 10.1007/s11548-021-02487-x

**Published:** 2021-09-09

**Authors:** Lina Felsner, Philipp Roser, Andreas Maier, Christian Riess

**Affiliations:** 1 Pattern Recognition Lab, Friedrich-Alexander Universität Erlangen-Nürnberg, Erlangen, Germany; 2 International Max Planck Research School - Physics of Light, Erlangen, Germany

**Keywords:** X-ray phase contrast imaging, Talbot–Lau interferometer, computed tomography, sensitivity correction, known operator learning

## Abstract

**Purpose:**

In Talbot–Lau X-ray phase contrast imaging, the measured phase value depends on the position of the object in the measurement setup. When imaging large objects, this may lead to inhomogeneous phase contributions within the object. These inhomogeneities introduce artifacts in tomographic reconstructions of the object.

**Methods:**

In this work, we compare recently proposed approaches to correct such reconstruction artifacts. We compare an iterative reconstruction algorithm, a known operator network and a U-net. The methods are qualitatively and quantitatively compared on the Shepp–Logan phantom and on the anatomy of a human abdomen. We also perform a dedicated experiment on the noise behavior of the methods.

**Results:**

All methods were able to reduce the specific artifacts in the reconstructions for the simulated and virtual real anatomy data. The results show method-specific residual errors that are indicative for the inherently different correction approaches. While all methods were able to correct the artifacts, we report a different noise behavior.

**Conclusion:**

The iterative reconstruction performs very well, but at the cost of a high runtime. The known operator network shows consistently a very competitive performance. The U-net performs slightly worse, but has the benefit that it is a general-purpose network that does not require special application knowledge.

## Introduction

**Fig. 1:**
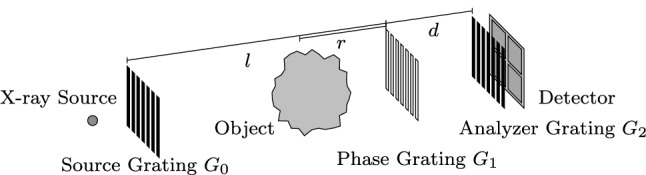
Schematic setup of a Talbot–Lau interferometer. *l* the distance between $G_0$ and $G_1$. *d* the distance between $G_1$ and $G_2$. *r* the distance between $G_1$ and a location in the object

X-ray phase contrast imaging provides high soft tissue contrast [16]. Similar to the attenuation, the phase shift can be modeled as a line integral along the X-ray beam. For thin samples, the measured phase shift $\phi$ is given as [15]

1 $$\begin{aligned} \phi = -k \int _{0}^t \delta (l) \,d l , \end{aligned}$$

where *k* is the wave number and $\delta$ is the phase shift along the line *l*. The Talbot–Lau interferometer (TLI) is a setup to obtain phase contrast images. Compared to other phase contrast measurement setups, the TLI has relatively mild system requirements and high robustness, which makes it suitable for several medical applications [8, 16] The TLI setup consists of three gratings placed between the X-ray source and the detector, as shown in Fig. 1. The source grating splits the beam into multiple slit sources, which makes it possible to operate the TLI with clinical X-ray sources. The phase grating imprints a phase modulation on to the incoming wave front. The analyzer grating samples the resulting fringe pattern. The phase signal is differential, obtained from a comparison of the fringe patterns with and without an object in the beam. The differential phase signal in a TLI is measured as

2 $$\begin{aligned} \varphi = \frac{\lambda d }{p_2}\frac{\partial \phi }{\partial t} , \end{aligned}$$

where $\lambda$ is the wave length, *d* the distance between gratings $G_1$ and $G_2$, and $p_2$ is the period of the analyzer grating. The measurement direction *t* is orthogonal to the grating bars, i.e., horizontally on the detector for vertical gratings.

Engelhard et al. [5] reported that the magnification of an object influences the strength of the differential phase signal in a TLI. They derived this effect theoretically and verified it experimentally. Donath et al. [4] confirmed those findings. They extended the theoretical formulation to an inverse setup and also verified the results experimentally. Thus, the measured phase shift $\varphi$ varies with the relative distance of the object to the gratings. We denote this effect as a position-dependent sensitivity function *S*(*r*)

3 $$\begin{aligned} S(r) = 2\pi \frac{d}{p_2} \cdot {\left\{ \begin{array}{ll} 1 + r/l, &{} \text {for objects between } G_0 \text { and } G_1\\ 1 - r/d, &{} \text {for objects between } G_1 \text { and } G_2 \end{array}\right. } , \nonumber \\ \end{aligned}$$

where *r* is the distance between the object position and $G_1$, *l* is the distance between $G_0$ and $G_1$, and *d* is again the distance between $G_1$ and $G_2$ [2]. These distances are also annotated in Fig. 1. A special case occurs for imaging with a parallel beam and with the object between $G_0$ and $G_1$. Then, *l* converges toward infinity and the sensitivity is constant.

Conversely, for a fan beam or an object between $G_1$ and $G_2$, the position-dependent sensitivity must to be considered to correctly determine the total phase shift. The phase shift of thin objects can be corrected with a global scaling factor. However, such a global correction is not applicable to thicker objects. In this case, the signal strength varies within the object. More specifically, the signal strength increases for voxels that are closer to $G_1$, even if the whole object consists of only one single material.

In particular, the position-dependent sensitivity complicates the tomographic reconstruction of the differential phase: Standard X-ray tomography is built on the basic assumption that the reconstructed quantity is constant under all projections [14]. However, if a thick object is imaged in a TLI tomography setup, one object location exhibits a stronger signal under rotation angles where the location is closer to $G_1$, and a weaker signal under rotation angles where the location is further away from $G_1$.

**Fig. 2:**
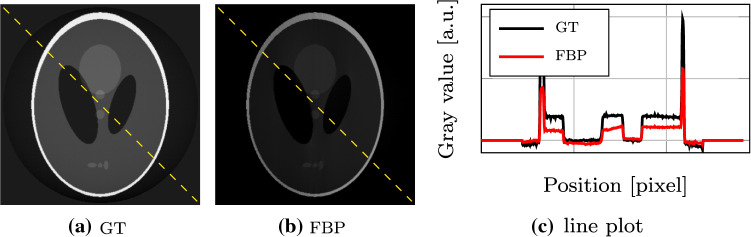
Ground-truth (GT) reconstruction of the Shepp–Logan phantom and filtered back-projection (FBP) reconstruction after a sensitivity weighted forward projection. Both images are windowed from 0 to 1 [a.u.]. The line plot follows the yellow dashed line

One possibility to address this issue is to use the position-dependent sensitivity from Eqn. 3 in a position- and angle-dependent function for tomography [2, 7]. Chabior were first to investigate this task [2]. They formulated the 2-D projection at a tomographic angle $\theta$ and the detector position *t* as

4 $$\begin{aligned} \varphi (\theta , t) = \frac{\partial }{\partial t} \int _{-\infty }^\infty S(r) \, \delta (t,r) \, d \mathbf {r} , \end{aligned}$$

where $\delta (t,r)$ encodes the object phase. The integration is performed along the ray direction $\mathbf {r}$, which depends on the tomographic angle $\theta$. They found a special scanning protocol, in which filtered back-projection (FBP) provides an artifact-free reconstruction: If the object is imaged over $2\pi$ with a parallel geometry, the linearity of the sensitivity function and symmetry of the imaging geometry average out the variations. The reconstruction shows in this case a homogeneous mean sensitivity, such that the sensitivity factor at the rotation center can be used to correct the reconstruction. However, when using a rotation angle other than $2\pi$ or a fan-beam geometry, the sensitivity function leads to severe reconstruction artifacts when using FBP. This is illustrated in Fig. 2. It shows a phantom on the left and a standard FBP reconstruction after projection with the sensitivity function from Eqn. 3 scaled between 0.1 and 0.9. The line plot on the right shows the reconstruction error, which results from the angle- and position-dependent sensitivity function.

Felsner et al. theoretically investigated a tomographic weighting function that depends on the voxel position and tomographic angle [7]. They described the reconstruction problem by integrating a weighting matrix that encodes the position- and angle-dependent function within the filtered back-projection. They show that the solution is ill-conditioned, such that there exists no exact solution. However, they derived an iterative reconstruction that approximates the ground truth very well.

Recently, Roser et al. have investigated a data-driven approach to the reconstruction task [19]. They propose a deep neural network that optimizes the reconstruction parameters for an included filtered back-projection (FBP) algorithm. They show on simulated data that this approach can greatly improve the reconstruction. Additionally, the results are interpretable, since the filtered back-projection is implemented as a known, fixed operator in the network.

While the approaches by Felsner et al. and Roser et al. are of very different nature, they are both specially designed to correct the position- and angle-dependent sensitivity function [7, 19]. Both approaches achieve high-quality reconstructions with few artifacts. However, the corrections are only evaluated on simple phantoms and do not compare the results to general-purpose state-of-the-art methods like U-net. In this work, we aim to close this gap. We compare the specialized methods by Felsner et al. [7], Roser et al. [19], and a general-purpose U-net [18] on a challenging set of test data.

## Methods

We first provide an overview over the methods and then describe the experimental data and protocol.

### Correction methods

**Iterative Reconstruction** Model-based iterative reconstruction is widely used to maintain image quality in cases of incomplete data. Felsner et al. used an algebraic least-squares reconstruction formulation [7], i.e.,

5 $$\begin{aligned} \min \frac{1}{2}\left| \left| {\mathbf {B}}{\mathbf {W}}\mathbf {x} - \mathbf {p} \right| \right| ^2_2 , \end{aligned}$$

where ${\mathbf {B}}$ is a block matrix containing the system matrices for each angle, ${\mathbf {W}}$ is a weighting matrix encoding the position- and angle-dependent factors, $\mathbf {x}$ is the vector representation of the object and $\mathbf {p}$ is the vector containing the projections. The optimization is done via gradient descent with the update rule

6 $$\begin{aligned} \mathbf {x}'= \mathbf {x} + \frac{p_i - \mathbf {b}_i {\mathbf {W}} \mathbf {x}}{\mathbf {b}_i {\mathbf {W}} {\mathbf {W}}^\top \mathbf {b}_i^\top } {\mathbf {W}}^\top \mathbf {b}_i^\top . \end{aligned}$$

Here, $p_i$ is one projection, $\mathbf {b}_i {\mathbf {W}} \mathbf {x}$ is the weighted forward projection, $p_i - \mathbf {b}_i {\mathbf {W}} \mathbf {x}$ is the gradient, and $\mathbf {b}_i {\mathbf {W}} {\mathbf {W}}^\top \mathbf {b}_i^\top$ is a normalization factor that contains the sum of squared weights along the ray. The optimization is regularized with Total Variation (TV) [22] to enable correct reconstructions for non-complete or distorted data on piecewise constant objects. This regularizer selects the image with sparsest gradient magnitude for the given data fidelity, which usually leads to very smooth images.

**Fig. 3:**
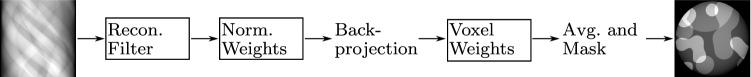
Known operator network by Roser et al. [19]. The network takes weighted projections as input. The output is a corrected reconstruction. The layers marked with a box are trainable

**Known Operator Network** The known operator theory learns efficient solutions for mathematical models with operands that are inefficient or unknown [13]. This allows interpretability of the learned quantity and possible insights into analytic solutions. Roser *et al.* model with known operators the sensitivity ramp in phase contrast imaging, by learning the inverse weighted Radon transform [19]. In the resulting neural network is each step of the pipeline modeled as a layer. This network optimizes the filters and the weighting layers. The input of the method are projections in form of a sinogram that contains line integrals of weighted phase values. The output is a corrected reconstruction. The pipeline is shown in Fig. 3.

**U-Net Network** Many ill-posed inverse problems highly benefit from general-purpose neural networks. The U-net is arguably the most popular multi-resolution neural network in medical image processing [18]. Previously, it was shown that the U-net is able to reduce reconstruction artifacts [9, 11]. In this work, we train and use a standard U-net to correct the artifacts in the reconstruction domain. We use the non-corrected reconstruction as an input and train the network to correct the artifacts.

### Experiments

**Fig. 4:**
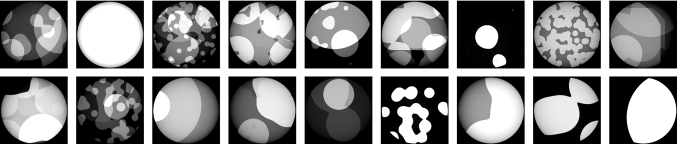
Example ground-truth data for training the neural network

We use a parallel beam geometry and define the sensitivity function as a linear ramp between 0.1 and 0.9 over a distance of 400 voxels. The trajectory uses 400 projections over an angular range of $\pi$. The data have a size of $400 \times 400$ voxels with a spacing of 1 mm. The detector consists of 400 pixels with a spacing of 1 mm. To directly use the iterative reconstruction and known operator network, we use direct (line integral) data instead of differential data.

As baseline method, we only scale the reconstruction by the sensitivity value at the iso-center, which does not remove inhomogeneous artifacts. The iterative reconstruction uses 500 iterations with an adaptive step size that starts at 0.75. The Lagrangian multiplier to control the amount of regularization started at 0.5 and could be adapted in the optimization process. The volume is initialized with the uncorrected FBP reconstruction. For the training of both neural networks, 1000 synthetic phantoms were used. Each phantom consists of a superposition of up to six randomly sized binary blobs with a gray value between zero and one. Figure 4 shows example inputs. For training and validation we use a 800/200 split. For the known operator network, we used the pipeline as described above. The filter in the known operator approach is initialized with the Ram–Lak filter [17]. The normalizing weights are initialized with ones, and the voxel weights are initialized with the mean sensitivity value. We use 100 epochs and optimize the parameters with respect to the mean absolute error (MAE) using stochastic gradient descent with adaptive moments [12] with a learning rate of $10^{-4}$. For the U-net architecture, we use a depth of 4 and 16 initial feature channels. Each block consist of two convolutional layers with a kernel size of $3\times 3$ and padding to preserve the dimensions of the feature map. The mean absolute error (MAE) is used as loss. The network is trained for 100 epochs with the Adam optimizer [12] using a learning rate of $10^{-4}$. We stopped the training when the validation loss did not improve for 20 epochs.

The methods are compared with three experiments. First, we perform a general comparison and in-depth evaluation on a simple phantom. Second, we evaluate the influence of different noise levels. Third, we evaluate the methods on a real human abdomen CT scan. These data originate from attenuation rather that a phase shift, but the real anatomical structures provide a much more realistic phantom for comparing the correction methods.

**Fig. 5:**
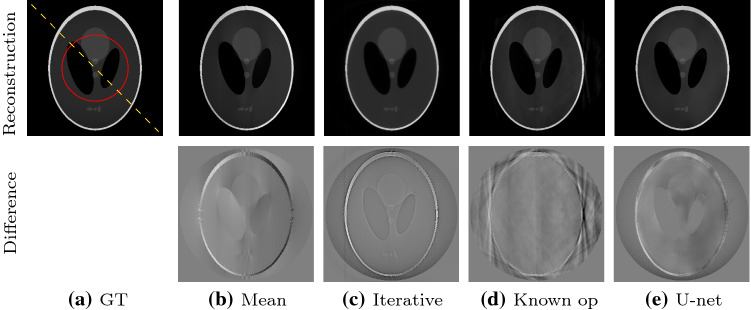
Reconstruction and difference images for all methods. The reconstructions are windowed from 0 to 1.2 [a.u]. The difference images are windowed from −0.25 to 0.25 [a.u.]

**General comparison** We evaluate all three methods on the Shepp–Logan phantom [21]. We show the reconstructions, the difference images to the ground truth and line plots. We also quantitatively evaluate the reconstructions quantitatively with the mean absolute error (MAE) and structural similarity (SSIM). The metrics are evaluated on the region of interest shown in Fig. 5a.

**Noise analysis** To evaluate the methods on noisy data, we simulate Poisson noise corresponding to photon counts ranging from 10000 to 100000 on the sensitivity-weighted line integral data. We show the reconstructions for the lowest and highest photon counts, and evaluate the reconstructions quantitatively with the peak signal-to-noise ratio (PSNR) and mean average percentage error (MAPE) compared to the noise-free ground truth.

**Real Data** To evaluate the behavior of the algorithms on real data, we used an human abdomen CT scan from the Cancer Imaging Archive [3, 20]. The data are normalized and resampled, and the sensitivity function is applied as described above. We show the reconstructed images and the difference image to the ground truth.

## Results

**General comparison** Figure 5 shows the reconstructions and difference images for all methods. All reconstructions are close to the ground truth. However, the difference images in the second row exhibit method-specific artifacts. The difference image for the mean correction shows a gradient from left to right. The difference image for the iterative reconstruction shows constant deviations per material. The reconstruction with the known operator network mainly shows streaking artifacts outside the object. The corrected reconstruction with a U-net clearly shows patch-wise artifacts.

**Fig. 6:**
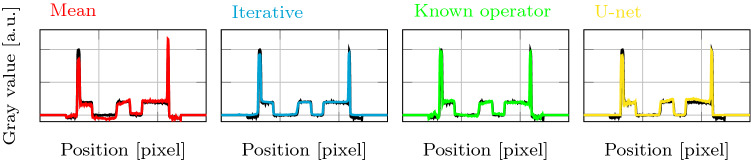
Line plots along the yellow dashed line in 5a. The ground truth is shown in black for each subplot

Figure 6 shows the line plots for the reconstructions of all methods along the yellow dashed line in Fig. 5a. The mean corrected version (red, leftmost) shows corrupted gray values. The signal is too low on the first half and too high on the second half. In particular, the ramp artifact in the object center is prominent. The line plot through the iterative reconstruction (blue, left) shows a good reconstruction. Although the main peaks are slightly too low, the result is smoother than the ground truth but very close to it. The reconstruction with the known operator network (green, right) is also close to the ground truth, including the heights of the peaks. However, some artifacts are visible inside the object and the ramp artifact in the center is not fully corrected. The reconstruction with the U-net (yellow, rightmost) is generally good, but the left peak is slightly underestimated, the object structure in the center shows a ramp artifact toward higher gray values, and the values on the right part of the object are slightly too high.

**Table 1:** Mean absolute error (MAE) and structural similarity (SSIM) for all methods. We evaluated the reconstruction in the circular region of interest marked in red in Fig. 5a

Error	FBP	mean	Iterative	known op	u-net
MAE	0.0748	0.0213	0.0080	0.0109	0.0171
SSIM	0.6438	0.9781	0.9953	0.9934	0.9837

The mean absolute error (MAE) and structural similarity (SSIM) index are reported in Table 1. Both metrics are considerably improved by all correction methods. The iterative reconstruction exhibits the lowest MAE, followed by the known operator network and U-net. The structural similarity (SSIM) for all three methods is in a similar order of magnitude: The SSIM for the iterative and known operator reconstruction differs only in the third digit after the decimal point, U-net performs is slightly worse.

**Fig. 7:**
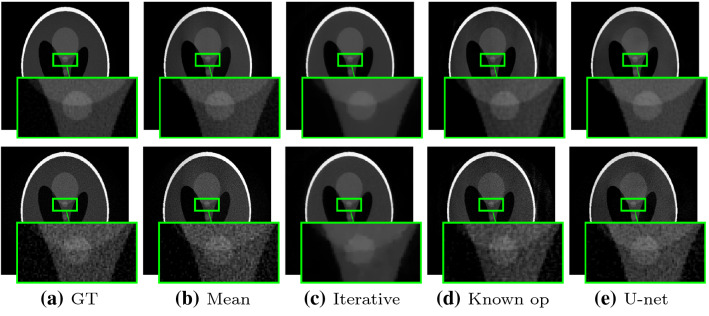
Reconstructions of all methods with noise. Top row: photon count of 1,00,000. Bottom row: photon count of 10,000. All reconstructions are windowed between 0 and 1 [a.u.]

**Fig. 8:**
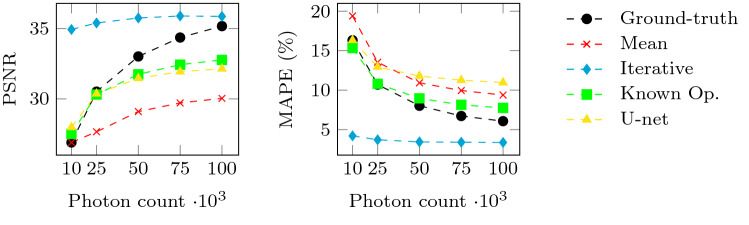
Peak signal-to-noise ratio (PSNR) and mean absolute percentage error (MAPE) for all methods over different noise levels

**Noise analysis** Figure 7 shows the reconstructions for the lowest and highest noise level in the test set. All methods are able to reconstruct the object at all noise levels. However, the details in the magnified area show slightly different noise characteristics. The iterative reconstruction results in a smooth object, which is expected and mainly due to the TV regularization. The known operator and U-net exhibit similarly strong noise as the ground-truth reconstruction. Interestingly, the noise with the U-net correction is finer compared to the reconstruction with the known operator network, while the noise in the known operator reconstructions is closer to the noisy ground truth.

Figure 8 shows the quantitative evaluation of the noise levels for PSNR (left) and MAPE (right). The PSNR of the ground truth (black) increases with the number of photons. At a photon count of 10000, The PSNR of mean corrected reconstruction equals the ground truth at 10000 photons, but the curve flattens with increasing photon counts. The PSNR of the iterative reconstruction is almost constant and higher than the ground-truth value for all noise levels, since the smoothing TV regularization fits very well to the smooth phantom. The PSNR for the reconstructions with the known operator network and the U-net are very similar. The U-net achieves a slightly higher PSNR for high noise, the known operator reconstruction performs slightly better for low noise. The MAPE of the ground truth decreases with an increasing number of photons. The mean corrected reconstruction follows the ground truth, but with consistently higher MAPE. The MAPE of the iterative reconstruction achieves the lowest error for all noise levels. The known operator network slightly outperforms the ground truth, the mean correction and U-net for high noise at 10000 photons. For higher photon counts, it is slightly worse than the ground truth. The U-net reconstruction is close to the ground truth for high noise at 10000 photons. Interestingly, for low noise (e.g., at 100000 photons), U-net performs worst with the highest MAPE.

**Fig. 9:**
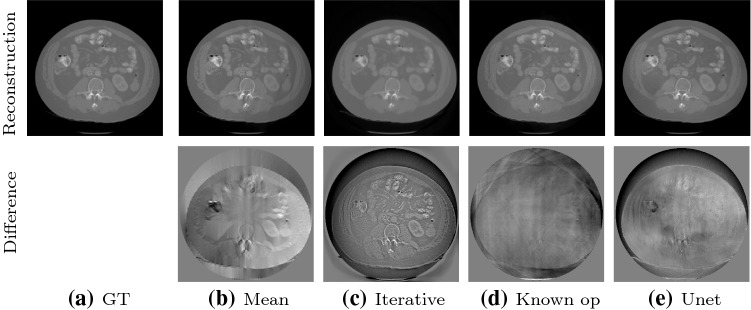
Reconstruction and difference images for all methods on a real human scan. The reconstructions are windowed from 0 to 1 [a.u.]. The difference images are windowed from −0.1 to 0.1 [a.u.]

**Real Anatomy Data** The real abdomen CT scan features far more complex structures than the Shepp–Logan phantom. The reconstructions for all methods and respective difference images are shown in Fig. 9. The ground truth is shown in Fig. 9a. The mean correction shows the same gradient artifacts as for the simulated data. The iterative reconstruction somewhat oversmooths due to the influence of the TV regularizer. All coarse objects structures are visible, the fine structures are smoothed out. This is also visible in the difference image. The reconstruction with the known operator network is very close to the ground truth, and the main artifacts are only outside the object. The reconstruction with the U-net introduces again patch artifacts, and it removes some fine structures. Differences are visible, especially in the structures on the top left.

## Discussion

The experiments showed that the corrected reconstructions of all three methods are superior to the baseline mean correction. Interestingly, we found that the residual errors are specific for each of the methods. In the mean corrected reconstruction, the values are systematically underestimated on one side and overestimated on the other side. This leads to the typical ramp artifact in the object center that can be observed in reconstruction with weighted projections. The direction of the ramp depends on the chosen trajectory. The iterative reconstruction clearly benefits from the TV regularization. The line plot shows an even smoother result than the ground truth, and the difference images confirm that the smoothing operator has significant impact. However, the iterative reconstruction has slight difficulties to correct high phase values at the object boundary. The difference image for the reconstruction with the known operator network shows only small deviations within the object and major artifacts outside the object. The line plot shows that the reconstruction has a similar noise level as the ground truth. In the object center, the ramp artifact is reduced but visible. The artifact correction with the general-purpose U-net architecture works overall quite well. The difference image and the line plot show uneven patch-wise artifacts. While the high intensity values on the left side of the object are underestimated, the right side of the object is largely overestimated. Interestingly, the deviation from the ground truth is even larger than with the mean correction. However, overall the correction with a U-net is overall preferable to the mean weight correction.

Furthermore, we qualitatively and quantitatively evaluated the impact of noise on the reconstructions. The reconstruction with the iterative method is very smooth. Reconstructions with the known operator network on noisy data are slightly worse than the ground truth, but consistently better than the mean corrected reconstruction. Only the U-net exhibits at low noise levels a slightly worse MAPE than the baseline.

The experiments on real data show that the methods also operate well on complex anatomic structures. We are surprised that all methods worked so well without changing any experimental parameters or training data. The artifacts agree with the artifacts on the Shepp–Logan phantom for all methods.

Overall, all three methods have specific advantages and disadvantages. U-net achieved a somewhat lower performance, but since it is a standard neural network architecture, it is the easiest to setup and run on this task. Also, additional skip connections for residual learning [11] or augmentation on noisy projections [9] can potentially improve the performance. Hence, the U-net is a good first choice without specialized tools. The known operator network provides an interpretable learning method with overall strong results. Integrating additional prior knowledge as in [1] could potentially further improve the reconstruction quality. The iterative reconstruction provides overall smooth, visually appealing reconstructions. The amount of regularization can be adjusted with the Lagrangian multiplier $\lambda$. However, besides the danger of oversmoothing, it must optimize the objective function for each new data, leading to run times that are orders of magnitude higher than for the neural networks.

Future work needs to investigate the adaption of the reconstruction algorithms to work on differential data. Alternatively, the differential TLI measurements could first be integrated to obtain projection images [8]. However, sharp edges that result in an infinite differential phase signal affect the quantitativeness of the differential phase signal and the integration [10, 23]. Also, noise in the differential data might influence the integration. Furthermore, the observed noise in the integrated images will depend on the used algorithm for the integration. Additionally, the currently limited grating size in a TLI might lead to truncation for large objects [6]. Future work should address a joint correction of the weighting function and truncation.

## Conclusion

We studied the reconstructions of three inherently different correction methods for the problem of a weighted projector. The results showed slight differences in the residual error in all experiments. The iterative reconstruction has the advantage of a smoothing TV regularization, but it has the highest runtime of all methods. The known operator network achieved very good reconstructions, but slightly amplified the noise in presence of low noise. The off-the-shelf U-net architecture worked surprisingly well. It is arguably the simplest to set up, at the expense of a slightly worse overall performance.
